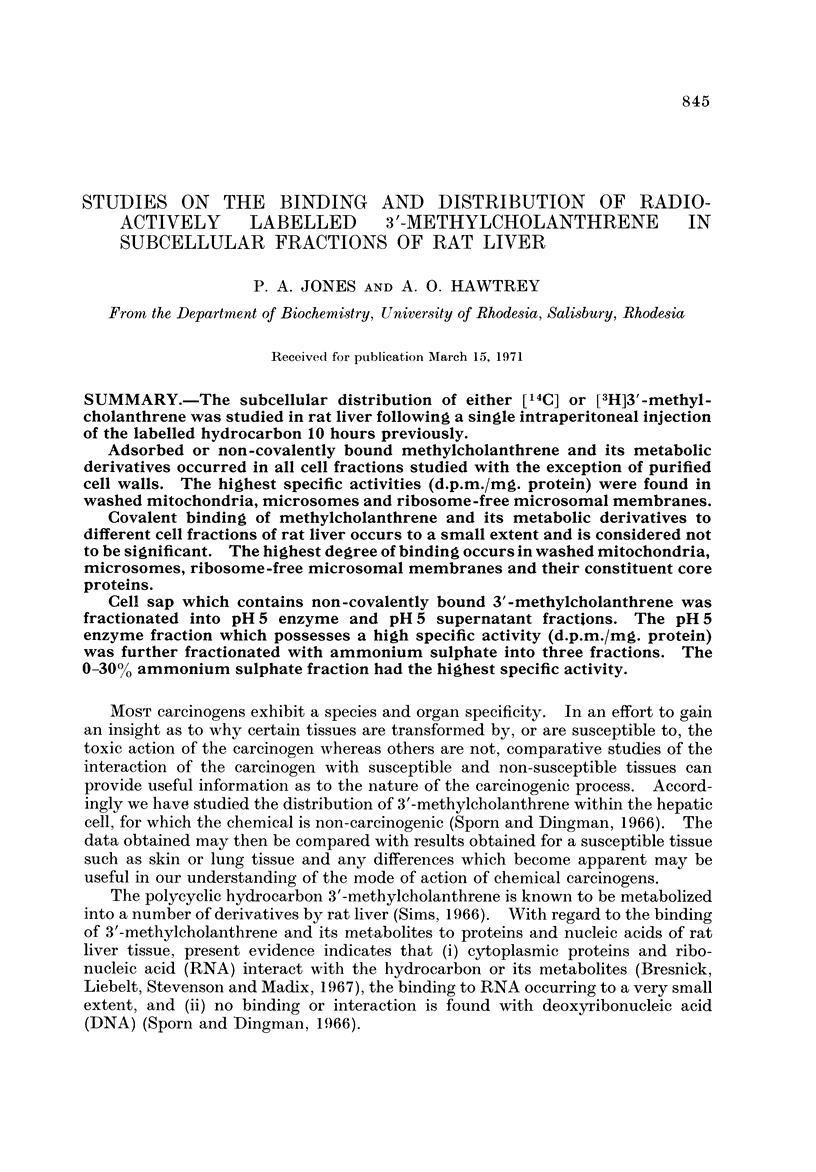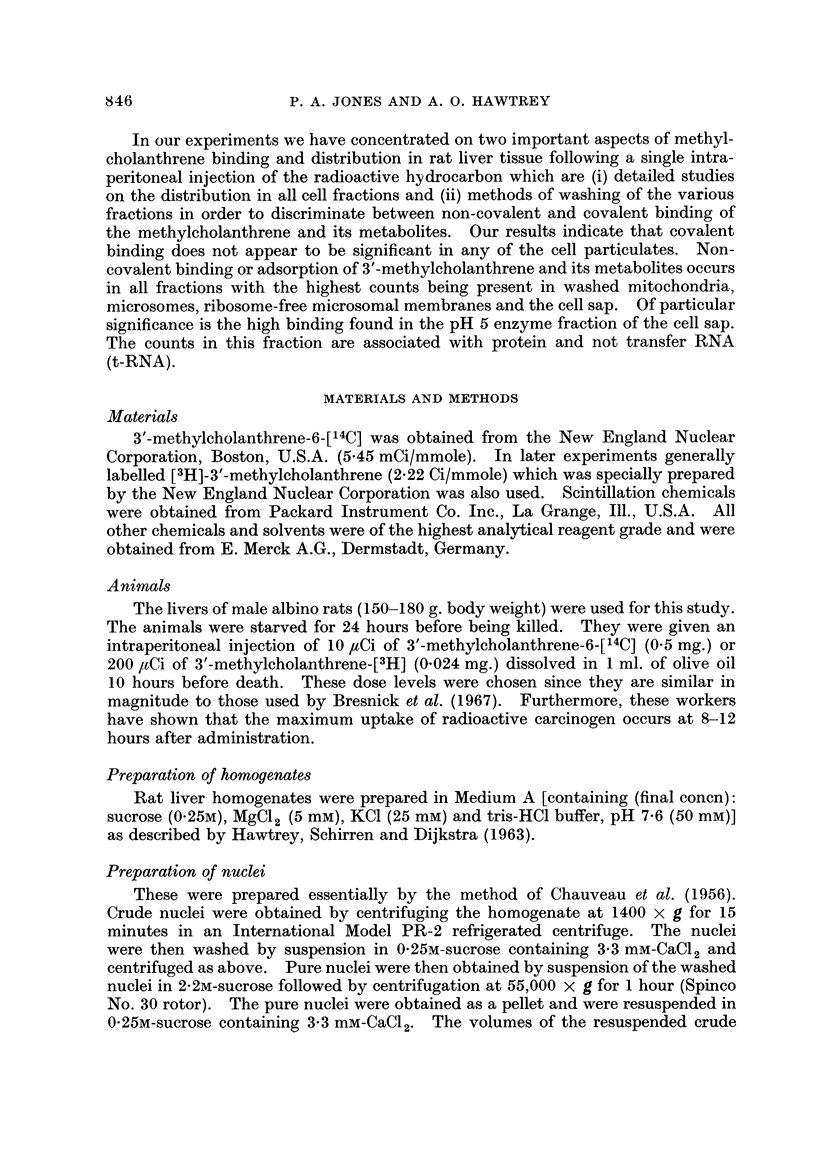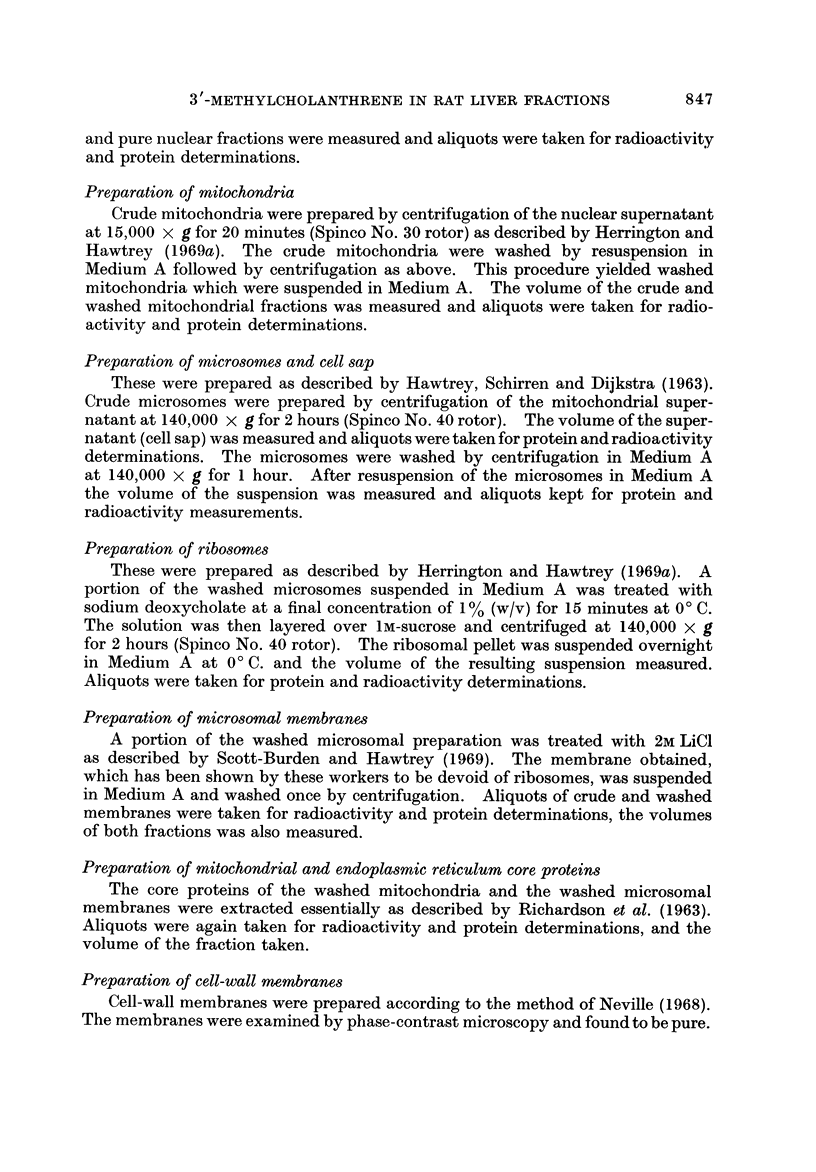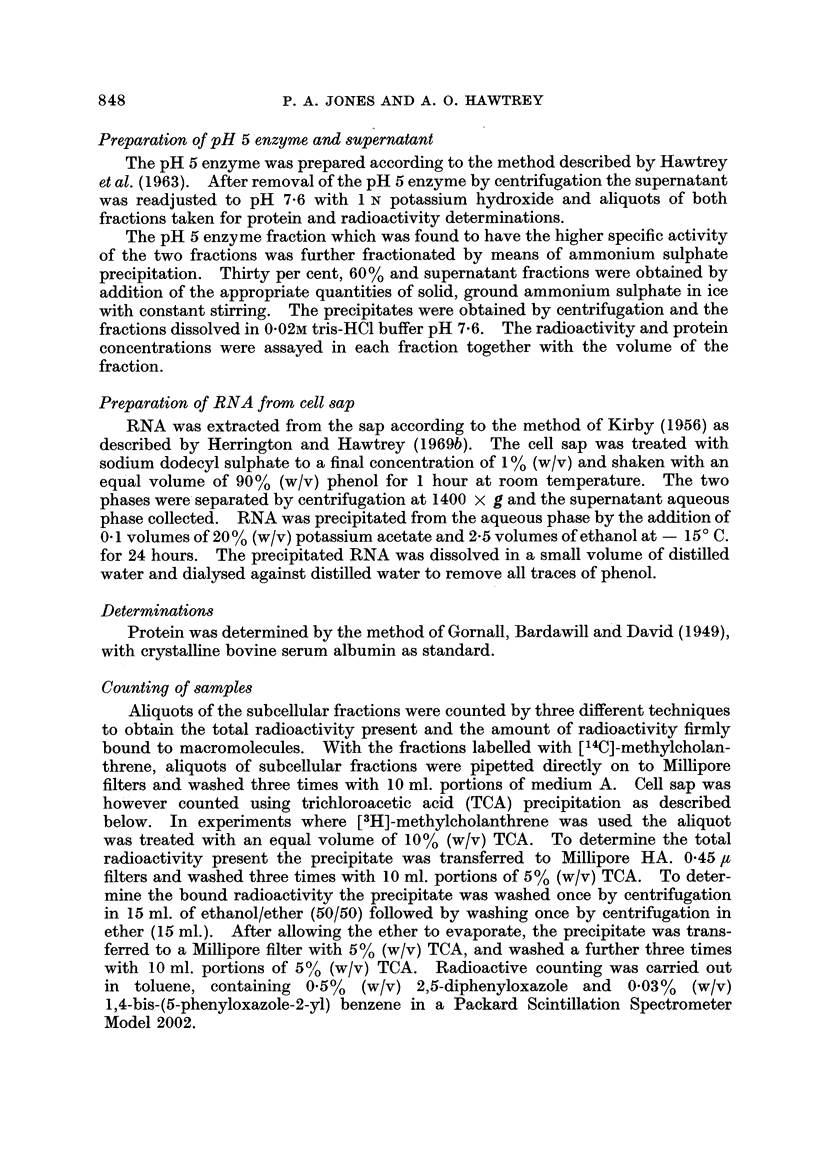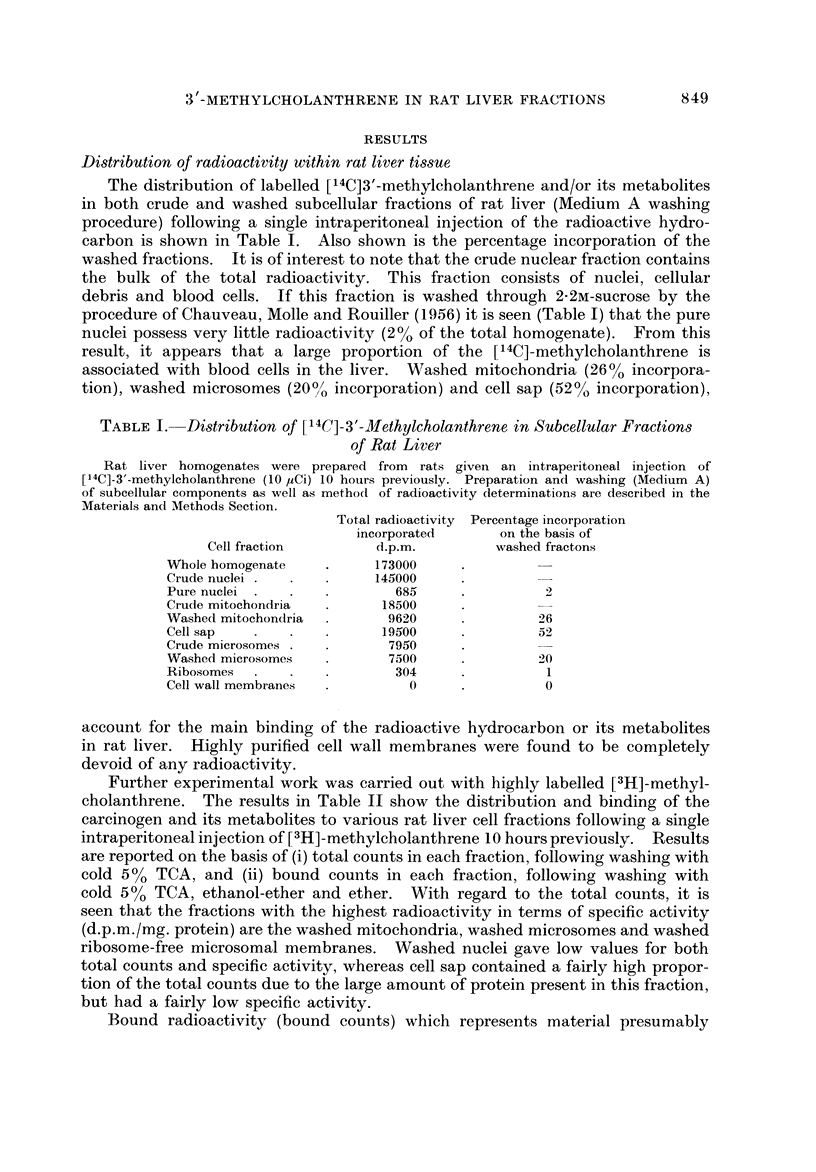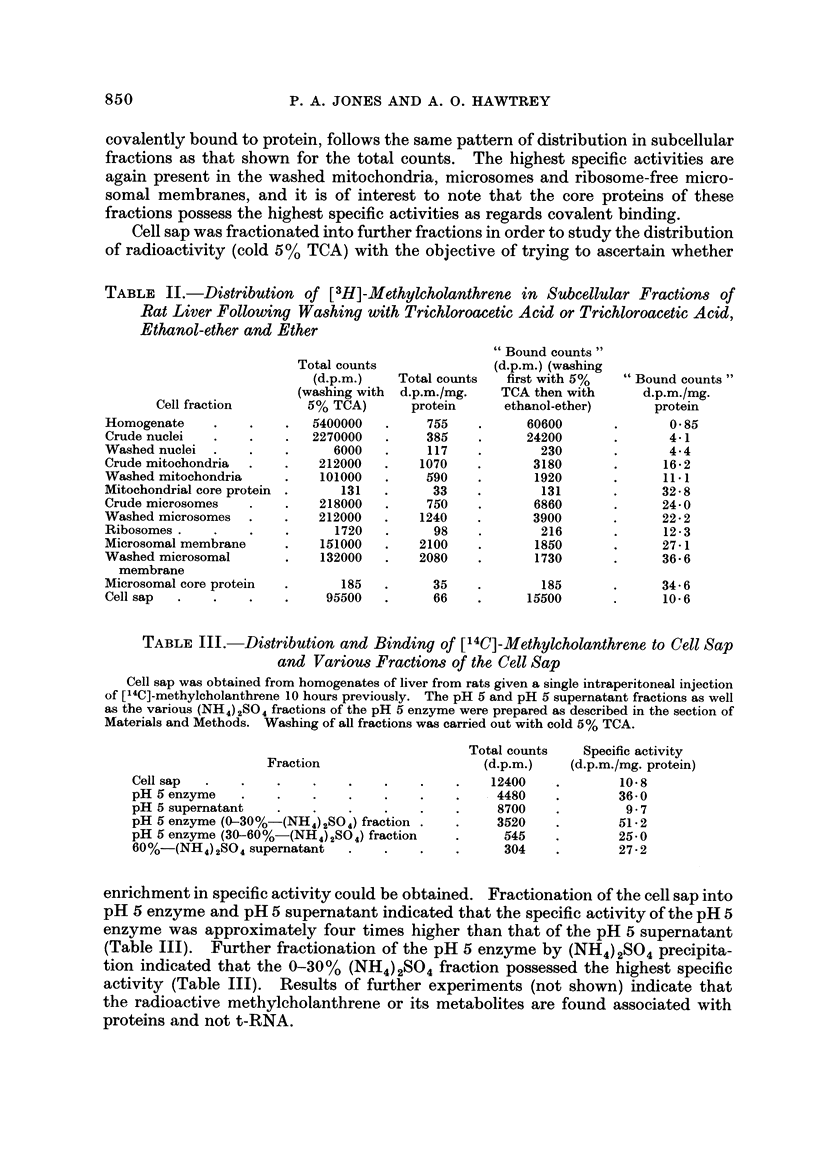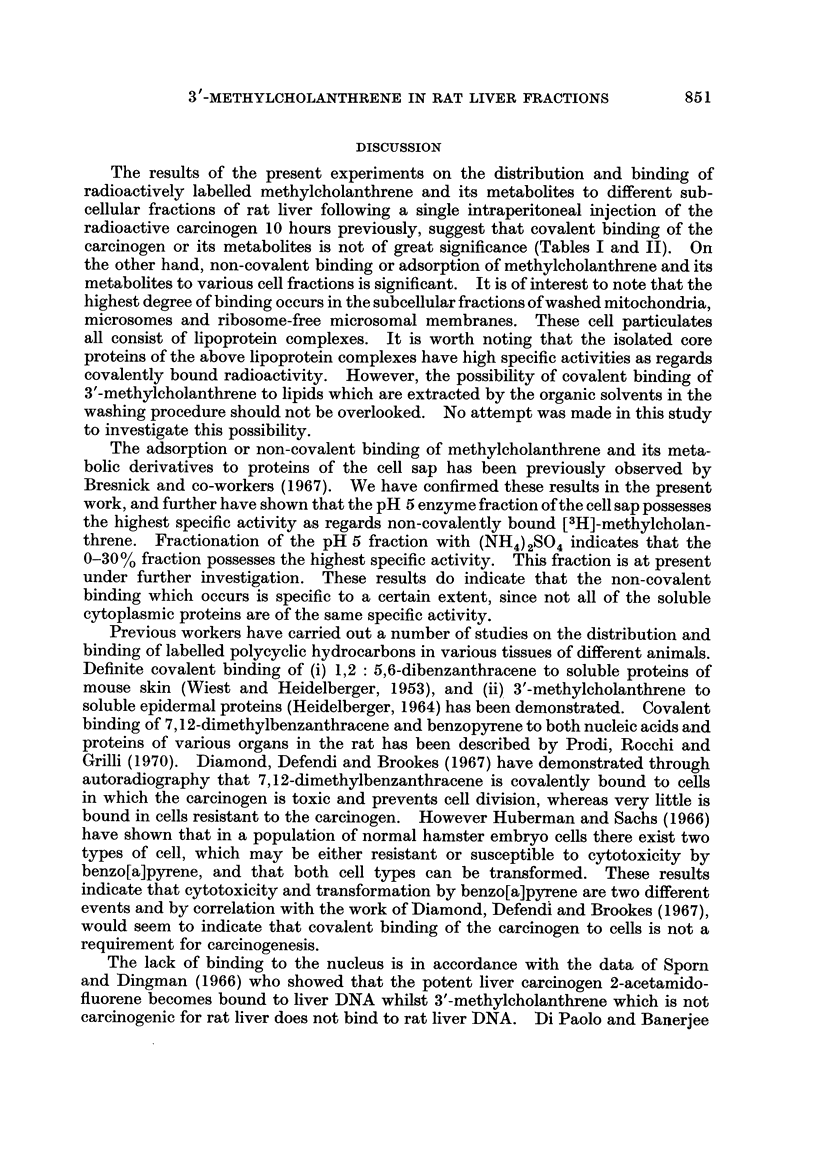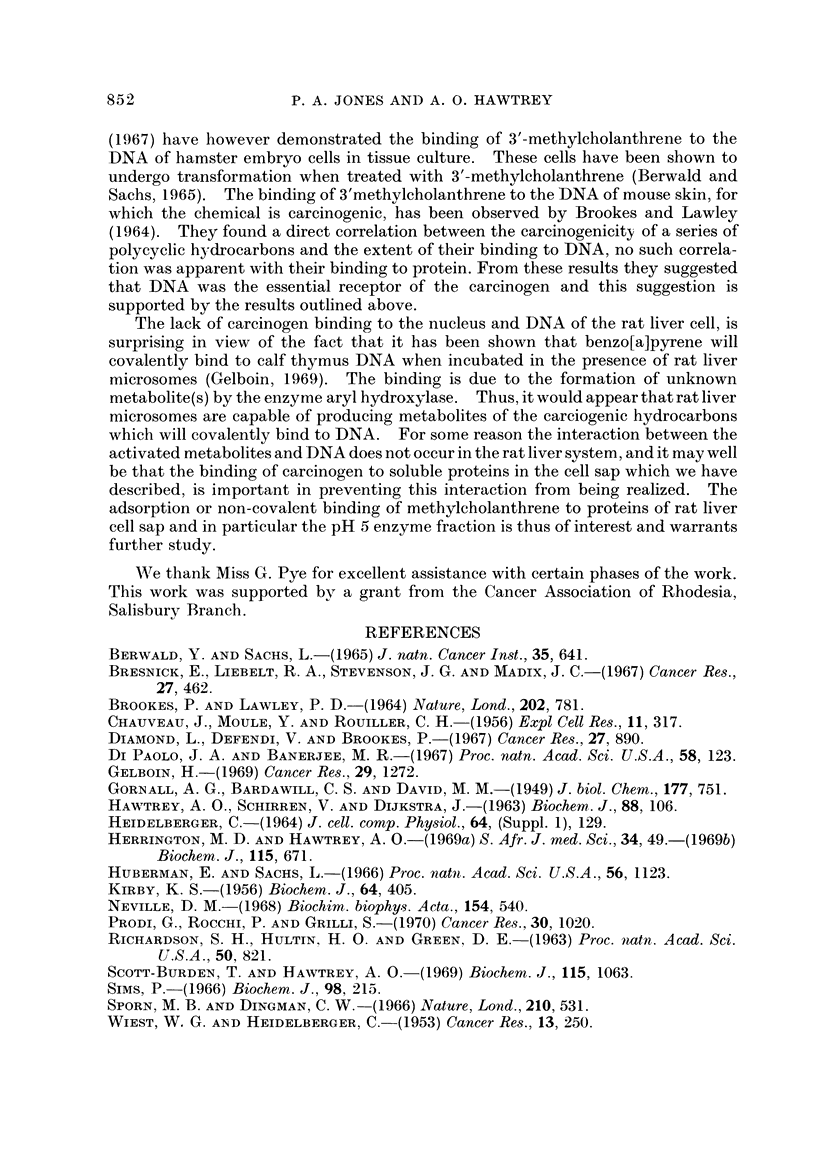# Studies on the Binding and Distribution of Radioactively Labelled 3′-Methylcholanthrene in Subcellular Fractions of Rat Liver

**DOI:** 10.1038/bjc.1971.98

**Published:** 1971-12

**Authors:** P. A. Jones, A. O. Hawtrey

## Abstract

The subcellular distribution of either [^14^C] or [^3^H]3′-methylcholanthrene was studied in rat liver following a single intraperitoneal injection of the labelled hydrocarbon 10 hours previously.

Adsorbed or non-covalently bound methylcholanthrene and its metabolic derivatives occurred in all cell fractions studied with the exception of purified cell walls. The highest specific activities (d.p.m./mg. protein) were found in washed mitochondria, microsomes and ribosome-free microsomal membranes.

Covalent binding of methylcholanthrene and its metabolic derivatives to different cell fractions of rat liver occurs to a small extent and is considered not to be significant. The highest degree of binding occurs in washed mitochondria, microsomes, ribosome-free microsomal membranes and their constituent core proteins.

Cell sap which contains non-covalently bound 3′-methylcholanthrene was fractionated into pH 5 enzyme and pH 5 supernatant fractions. The pH 5 enzyme fraction which possesses a high specific activity (d.p.m./mg. protein) was further fractionated with ammonium sulphate into three fractions. The 0-30% ammonium sulphate fraction had the highest specific activity.


### STUDIES ON THE BINDING AND DISTRIBUTION OF RADIO-ACTIVELY LABELLED 3′-METHYLCHOLANTHRENE IN SUBCELLULAR FRACTIONS OF RAT LIVER

P. A. JONES AND A. O. HAWTREY

*From the Department of Biochemistry, University of Rhodesia, Salisbury, Rhodesia*

Received for publication March 15, 1971

**SUMMARY.—The subcellular distribution of either [$^{14}$C] or [$^{3}$H]3′-methylcholanthrene was studied in rat liver following a single intraperitoneal injection of the labelled hydrocarbon 10 hours previously.**

**Adsorbed or non-covalently bound methylcholanthrene and its metabolic derivatives occurred in all cell fractions studied with the exception of purified cell walls. The highest specific activities (d.p.m./mg. protein) were found in washed mitochondria, microsomes and ribosome-free microsomal membranes.**

**Covalent binding of methylcholanthrene and its metabolic derivatives to different cell fractions of rat liver occurs to a small extent and is considered not to be significant. The highest degree of binding occurs in washed mitochondria, microsomes, ribosome-free microsomal membranes and their constituent core proteins.**

**Cell sap which contains non-covalently bound 3′-methylcholanthrene was fractionated into pH 5 enzyme and pH 5 supernatant fractions. The pH 5 enzyme fraction which possesses a high specific activity (d.p.m./mg. protein) was further fractionated with ammonium sulphate into three fractions. The 0–30% ammonium sulphate fraction had the highest specific activity.**

MOST carcinogens exhibit a species and organ specificity. In an effort to gain an insight as to why certain tissues are transformed by, or are susceptible to, the toxic action of the carcinogen whereas others are not, comparative studies of the interaction of the carcinogen with susceptible and non-susceptible tissues can provide useful information as to the nature of the carcinogenic process. Accordingly we have studied the distribution of 3′-methylcholanthrene within the hepatic cell, for which the chemical is non-carcinogenic (Sporn and Dingman, 1966). The data obtained may then be compared with results obtained for a susceptible tissue such as skin or lung tissue and any differences which become apparent may be useful in our understanding of the mode of action of chemical carcinogens.

The polycyclic hydrocarbon 3′-methylcholanthrene is known to be metabolized into a number of derivatives by rat liver (Sims, 1966). With regard to the binding of 3′-methylcholanthrene and its metabolites to proteins and nucleic acids of rat liver tissue, present evidence indicates that (i) cytoplasmic proteins and ribonucleic acid (RNA) interact with the hydrocarbon or its metabolites (Bresnick, Liebelt, Stevenson and Madix, 1967), the binding to RNA occurring to a very small extent, and (ii) no binding or interaction is found with deoxyribonucleic acid (DNA) (Sporn and Dingman, 1966).

In our experiments we have concentrated on two important aspects of methylcholanthrene binding and distribution in rat liver tissue following a single intraperitoneal injection of the radioactive hydrocarbon which are (i) detailed studies on the distribution in all cell fractions and (ii) methods of washing of the various fractions in order to discriminate between non-covalent and covalent binding of the methylcholanthrene and its metabolites. Our results indicate that covalent binding does not appear to be significant in any of the cell particulates. Non-covalent binding or adsorption of 3′-methylcholanthrene and its metabolites occurs in all fractions with the highest counts being present in washed mitochondria, microsomes, ribosome-free microsomal membranes and the cell sap. Of particular significance is the high binding found in the pH 5 enzyme fraction of the cell sap. The counts in this fraction are associated with protein and not transfer RNA (t-RNA).

### MATERIALS AND METHODS

### *Materials*

3′-methylcholanthrene-6-[$^{14}C$] was obtained from the New England Nuclear Corporation, Boston, U.S.A. (5·45 mCi/mmole). In later experiments generally labelled [$^{3}H$]-3′-methylcholanthrene (2·22 Ci/mmole) which was specially prepared by the New England Nuclear Corporation was also used. Scintillation chemicals were obtained from Packard Instrument Co. Inc., La Grange, Ill., U.S.A. All other chemicals and solvents were of the highest analytical reagent grade and were obtained from E. Merck A.G., Dermstadt, Germany.

### *Animals*

The livers of male albino rats (150–180 g. body weight) were used for this study. The animals were starved for 24 hours before being killed. They were given an intraperitoneal injection of 10 $\mu$Ci of 3′-methylcholanthrene-6-[$^{14}C$] (0·5 mg.) or 200 $\mu$Ci of 3′-methylcholanthrene-[$^{3}H$] (0·024 mg.) dissolved in 1 ml. of olive oil 10 hours before death. These dose levels were chosen since they are similar in magnitude to those used by Bresnick *et al.* (1967). Furthermore, these workers have shown that the maximum uptake of radioactive carcinogen occurs at 8–12 hours after administration.

### *Preparation of homogenates*

Rat liver homogenates were prepared in Medium A [containing (final concn): sucrose (0·25M), $MgCl_2$ (5 mM), KCl (25 mM) and tris-HCl buffer, pH 7·6 (50 mM)] as described by Hawtrey, Schirren and Dijkstra (1963).

### *Preparation of nuclei*

These were prepared essentially by the method of Chauveau *et al.* (1956). Crude nuclei were obtained by centrifuging the homogenate at 1400 × ***g*** for 15 minutes in an International Model PR-2 refrigerated centrifuge. The nuclei were then washed by suspension in 0·25M-sucrose containing 3·3 mM-$CaCl_2$ and centrifuged as above. Pure nuclei were then obtained by suspension of the washed nuclei in 2·2M-sucrose followed by centrifugation at 55,000 × ***g*** for 1 hour (Spinco No. 30 rotor). The pure nuclei were obtained as a pellet and were resuspended in 0·25M-sucrose containing 3·3 mM-$CaCl_2$. The volumes of the resuspended crude and pure nuclear fractions were measured and aliquots were taken for radioactivity and protein determinations.

*Preparation of mitochondria*

Crude mitochondria were prepared by centrifugation of the nuclear supernatant at 15,000 × *g* for 20 minutes (Spinco No. 30 rotor) as described by Herrington and Hawtrey (1969*a*). The crude mitochondria were washed by resuspension in Medium A followed by centrifugation as above. This procedure yielded washed mitochondria which were suspended in Medium A. The volume of the crude and washed mitochondrial fractions was measured and aliquots were taken for radioactivity and protein determinations.

*Preparation of microsomes and cell sap*

These were prepared as described by Hawtrey, Schirren and Dijkstra (1963). Crude microsomes were prepared by centrifugation of the mitochondrial supernatant at 140,000 × *g* for 2 hours (Spinco No. 40 rotor). The volume of the supernatant (cell sap) was measured and aliquots were taken for protein and radioactivity determinations. The microsomes were washed by centrifugation in Medium A at 140,000 × *g* for 1 hour. After resuspension of the microsomes in Medium A the volume of the suspension was measured and aliquots kept for protein and radioactivity measurements.

*Preparation of ribosomes*

These were prepared as described by Herrington and Hawtrey (1969*a*). A portion of the washed microsomes suspended in Medium A was treated with sodium deoxycholate at a final concentration of 1% (w/v) for 15 minutes at 0° C. The solution was then layered over 1M-sucrose and centrifuged at 140,000 × *g* for 2 hours (Spinco No. 40 rotor). The ribosomal pellet was suspended overnight in Medium A at 0° C. and the volume of the resulting suspension measured. Aliquots were taken for protein and radioactivity determinations.

*Preparation of microsomal membranes*

A portion of the washed microsomal preparation was treated with 2M LiCl as described by Scott-Burden and Hawtrey (1969). The membrane obtained, which has been shown by these workers to be devoid of ribosomes, was suspended in Medium A and washed once by centrifugation. Aliquots of crude and washed membranes were taken for radioactivity and protein determinations, the volumes of both fractions was also measured.

*Preparation of mitochondrial and endoplasmic reticulum core proteins*

The core proteins of the washed mitochondria and the washed microsomal membranes were extracted essentially as described by Richardson *et al.* (1963). Aliquots were again taken for radioactivity and protein determinations, and the volume of the fraction taken.

*Preparation of cell-wall membranes*

Cell-wall membranes were prepared according to the method of Neville (1968). The membranes were examined by phase-contrast microscopy and found to be pure.

*Preparation of pH 5 enzyme and supernatant*

The pH 5 enzyme was prepared according to the method described by Hawtrey *et al.* (1963). After removal of the pH 5 enzyme by centrifugation the supernatant was readjusted to pH 7·6 with 1 N potassium hydroxide and aliquots of both fractions taken for protein and radioactivity determinations.

The pH 5 enzyme fraction which was found to have the higher specific activity of the two fractions was further fractionated by means of ammonium sulphate precipitation. Thirty per cent, 60% and supernatant fractions were obtained by addition of the appropriate quantities of solid, ground ammonium sulphate in ice with constant stirring. The precipitates were obtained by centrifugation and the fractions dissolved in 0·02M tris-HCl buffer pH 7·6. The radioactivity and protein concentrations were assayed in each fraction together with the volume of the fraction.

*Preparation of RNA from cell sap*

RNA was extracted from the sap according to the method of Kirby (1956) as described by Herrington and Hawtrey (1969*b*). The cell sap was treated with sodium dodecyl sulphate to a final concentration of 1% (w/v) and shaken with an equal volume of 90% (w/v) phenol for 1 hour at room temperature. The two phases were separated by centrifugation at 1400 × *g* and the supernatant aqueous phase collected. RNA was precipitated from the aqueous phase by the addition of 0·1 volumes of 20% (w/v) potassium acetate and 2·5 volumes of ethanol at − 15° C. for 24 hours. The precipitated RNA was dissolved in a small volume of distilled water and dialysed against distilled water to remove all traces of phenol.

*Determinations*

Protein was determined by the method of Gornall, Bardawill and David (1949), with crystalline bovine serum albumin as standard.

*Counting of samples*

Aliquots of the subcellular fractions were counted by three different techniques to obtain the total radioactivity present and the amount of radioactivity firmly bound to macromolecules. With the fractions labelled with [$^{14}$C]-methylcholanthrene, aliquots of subcellular fractions were pipetted directly on to Millipore filters and washed three times with 10 ml. portions of medium A. Cell sap was however counted using trichloroacetic acid (TCA) precipitation as described below. In experiments where [$^{3}$H]-methylcholanthrene was used the aliquot was treated with an equal volume of 10% (w/v) TCA. To determine the total radioactivity present the precipitate was transferred to Millipore HA. 0·45 $\mu$ filters and washed three times with 10 ml. portions of 5% (w/v) TCA. To determine the bound radioactivity the precipitate was washed once by centrifugation in 15 ml. of ethanol/ether (50/50) followed by washing once by centrifugation in ether (15 ml.). After allowing the ether to evaporate, the precipitate was transferred to a Millipore filter with 5% (w/v) TCA, and washed a further three times with 10 ml. portions of 5% (w/v) TCA. Radioactive counting was carried out in toluene, containing 0·5% (w/v) 2,5-diphenyloxazole and 0·03% (w/v) 1,4-bis-(5-phenyloxazole-2-yl) benzene in a Packard Scintillation Spectrometer Model 2002.

### RESULTS

### *Distribution of radioactivity within rat liver tissue*

The distribution of labelled [$^{14}$C]3′-methylcholanthrene and/or its metabolites in both crude and washed subcellular fractions of rat liver (Medium A washing procedure) following a single intraperitoneal injection of the radioactive hydrocarbon is shown in Table I. Also shown is the percentage incorporation of the washed fractions. It is of interest to note that the crude nuclear fraction contains the bulk of the total radioactivity. This fraction consists of nuclei, cellular debris and blood cells. If this fraction is washed through 2·2M-sucrose by the procedure of Chauveau, Molle and Rouiller (1956) it is seen (Table I) that the pure nuclei possess very little radioactivity (2% of the total homogenate). From this result, it appears that a large proportion of the [$^{14}$C]-methylcholanthrene is associated with blood cells in the liver. Washed mitochondria (26% incorporation), washed microsomes (20% incorporation) and cell sap (52% incorporation),

TABLE I.—*Distribution of* [$^{14}$*C*]-3′-*Methylcholanthrene in Subcellular Fractions of Rat Liver*

Rat liver homogenates were prepared from rats given an intraperitoneal injection of [$^{14}$C]-3′-methylcholanthrene (10 $\mu$Ci) 10 hours previously. Preparation and washing (Medium A) of subcellular components as well as method of radioactivity determinations are described in the Materials and Methods Section.

| Cell fraction | Total radioactivity incorporated d.p.m. | Percentage incorporation on the basis of washed fractons |
|---|---|---|
| Whole homogenate | 173000 | — |
| Crude nuclei | 145000 | — |
| Pure nuclei | 685 | 2 |
| Crude mitochondria | 18500 | — |
| Washed mitochondria | 9620 | 26 |
| Cell sap | 19500 | 52 |
| Crude microsomes | 7950 | — |
| Washed microsomes | 7500 | 20 |
| Ribosomes | 304 | 1 |
| Cell wall membranes | 0 | 0 |

account for the main binding of the radioactive hydrocarbon or its metabolites in rat liver. Highly purified cell wall membranes were found to be completely devoid of any radioactivity.

Further experimental work was carried out with highly labelled [$^{3}$H]-methylcholanthrene. The results in Table II show the distribution and binding of the carcinogen and its metabolites to various rat liver cell fractions following a single intraperitoneal injection of [$^{3}$H]-methylcholanthrene 10 hours previously. Results are reported on the basis of (i) total counts in each fraction, following washing with cold 5% TCA, and (ii) bound counts in each fraction, following washing with cold 5% TCA, ethanol-ether and ether. With regard to the total counts, it is seen that the fractions with the highest radioactivity in terms of specific activity (d.p.m./mg. protein) are the washed mitochondria, washed microsomes and washed ribosome-free microsomal membranes. Washed nuclei gave low values for both total counts and specific activity, whereas cell sap contained a fairly high proportion of the total counts due to the large amount of protein present in this fraction, but had a fairly low specific activity.

Bound radioactivity (bound counts) which represents material presumably covalently bound to protein, follows the same pattern of distribution in subcellular fractions as that shown for the total counts. The highest specific activities are again present in the washed mitochondria, microsomes and ribosome-free microsomal membranes, and it is of interest to note that the core proteins of these fractions possess the highest specific activities as regards covalent binding.

Cell sap was fractionated into further fractions in order to study the distribution of radioactivity (cold 5% TCA) with the objective of trying to ascertain whether

TABLE II.—*Distribution of [$^3H$]-Methylcholanthrene in Subcellular Fractions of Rat Liver Following Washing with Trichloroacetic Acid or Trichloroacetic Acid, Ethanol-ether and Ether*

| Cell fraction | Total counts (d.p.m.) (washing with 5% TCA) | Total counts d.p.m./mg. protein | " Bound counts " (d.p.m.) (washing first with 5% TCA then with ethanol-ether) | " Bound counts " d.p.m./mg. protein |
|---|---|---|---|---|
| Homogenate | 5400000 | 755 | 60600 | 0·85 |
| Crude nuclei | 2270000 | 385 | 24200 | 4·1 |
| Washed nuclei | 6000 | 117 | 230 | 4·4 |
| Crude mitochondria | 212000 | 1070 | 3180 | 16·2 |
| Washed mitochondria | 101000 | 590 | 1920 | 11·1 |
| Mitochondrial core protein | 131 | 33 | 131 | 32·8 |
| Crude microsomes | 218000 | 750 | 6860 | 24·0 |
| Washed microsomes | 212000 | 1240 | 3900 | 22·2 |
| Ribosomes | 1720 | 98 | 216 | 12·3 |
| Microsomal membrane | 151000 | 2100 | 1850 | 27·1 |
| Washed microsomal membrane | 132000 | 2080 | 1730 | 36·6 |
| Microsomal core protein | 185 | 35 | 185 | 34·6 |
| Cell sap | 95500 | 66 | 15500 | 10·6 |

TABLE III.—*Distribution and Binding of [$^{14}C$]-Methylcholanthrene to Cell Sap and Various Fractions of the Cell Sap*

Cell sap was obtained from homogenates of liver from rats given a single intraperitoneal injection of [$^{14}$C]-methylcholanthrene 10 hours previously. The pH 5 and pH 5 supernatant fractions as well as the various $(NH_4)_2SO_4$ fractions of the pH 5 enzyme were prepared as described in the section of Materials and Methods. Washing of all fractions was carried out with cold 5% TCA.

| Fraction | Total counts (d.p.m.) | Specific activity (d.p.m./mg. protein) |
|---|---|---|
| Cell sap | 12400 | 10·8 |
| pH 5 enzyme | 4480 | 36·0 |
| pH 5 supernatant | 8700 | 9·7 |
| pH 5 enzyme (0–30%—$(NH_4)_2SO_4$) fraction | 3520 | 51·2 |
| pH 5 enzyme (30–60%—$(NH_4)_2SO_4$) fraction | 545 | 25·0 |
| 60%—$(NH_4)_2SO_4$ supernatant | 304 | 27·2 |

enrichment in specific activity could be obtained. Fractionation of the cell sap into pH 5 enzyme and pH 5 supernatant indicated that the specific activity of the pH 5 enzyme was approximately four times higher than that of the pH 5 supernatant (Table III). Further fractionation of the pH 5 enzyme by $(NH_4)_2SO_4$ precipitation indicated that the 0–30% $(NH_4)_2SO_4$ fraction possessed the highest specific activity (Table III). Results of further experiments (not shown) indicate that the radioactive methylcholanthrene or its metabolites are found associated with proteins and not t-RNA.

### DISCUSSION

The results of the present experiments on the distribution and binding of radioactively labelled methylcholanthrene and its metabolites to different subcellular fractions of rat liver following a single intraperitoneal injection of the radioactive carcinogen 10 hours previously, suggest that covalent binding of the carcinogen or its metabolites is not of great significance (Tables I and II). On the other hand, non-covalent binding or adsorption of methylcholanthrene and its metabolites to various cell fractions is significant. It is of interest to note that the highest degree of binding occurs in the subcellular fractions of washed mitochondria, microsomes and ribosome-free microsomal membranes. These cell particulates all consist of lipoprotein complexes. It is worth noting that the isolated core proteins of the above lipoprotein complexes have high specific activities as regards covalently bound radioactivity. However, the possibility of covalent binding of 3'-methylcholanthrene to lipids which are extracted by the organic solvents in the washing procedure should not be overlooked. No attempt was made in this study to investigate this possibility.

The adsorption or non-covalent binding of methylcholanthrene and its metabolic derivatives to proteins of the cell sap has been previously observed by Bresnick and co-workers (1967). We have confirmed these results in the present work, and further have shown that the pH 5 enzyme fraction of the cell sap possesses the highest specific activity as regards non-covalently bound [$^3$H]-methylcholanthrene. Fractionation of the pH 5 fraction with $(NH_4)_2SO_4$ indicates that the 0–30% fraction possesses the highest specific activity. This fraction is at present under further investigation. These results do indicate that the non-covalent binding which occurs is specific to a certain extent, since not all of the soluble cytoplasmic proteins are of the same specific activity.

Previous workers have carried out a number of studies on the distribution and binding of labelled polycyclic hydrocarbons in various tissues of different animals. Definite covalent binding of (i) 1,2 : 5,6-dibenzanthracene to soluble proteins of mouse skin (Wiest and Heidelberger, 1953), and (ii) 3'-methylcholanthrene to soluble epidermal proteins (Heidelberger, 1964) has been demonstrated. Covalent binding of 7,12-dimethylbenzanthracene and benzopyrene to both nucleic acids and proteins of various organs in the rat has been described by Prodi, Rocchi and Grilli (1970). Diamond, Defendi and Brookes (1967) have demonstrated through autoradiography that 7,12-dimethylbenzanthracene is covalently bound to cells in which the carcinogen is toxic and prevents cell division, whereas very little is bound in cells resistant to the carcinogen. However Huberman and Sachs (1966) have shown that in a population of normal hamster embryo cells there exist two types of cell, which may be either resistant or susceptible to cytotoxicity by benzo[a]pyrene, and that both cell types can be transformed. These results indicate that cytotoxicity and transformation by benzo[a]pyrene are two different events and by correlation with the work of Diamond, Defendi and Brookes (1967), would seem to indicate that covalent binding of the carcinogen to cells is not a requirement for carcinogenesis.

The lack of binding to the nucleus is in accordance with the data of Sporn and Dingman (1966) who showed that the potent liver carcinogen 2-acetamidofluorene becomes bound to liver DNA whilst 3'-methylcholanthrene which is not carcinogenic for rat liver does not bind to rat liver DNA. Di Paolo and Banerjee

(1967) have however demonstrated the binding of 3′-methylcholanthrene to the DNA of hamster embryo cells in tissue culture. These cells have been shown to undergo transformation when treated with 3′-methylcholanthrene (Berwald and Sachs, 1965). The binding of 3′methylcholanthrene to the DNA of mouse skin, for which the chemical is carcinogenic, has been observed by Brookes and Lawley (1964). They found a direct correlation between the carcinogenicity of a series of polycyclic hydrocarbons and the extent of their binding to DNA, no such correlation was apparent with their binding to protein. From these results they suggested that DNA was the essential receptor of the carcinogen and this suggestion is supported by the results outlined above.

The lack of carcinogen binding to the nucleus and DNA of the rat liver cell, is surprising in view of the fact that it has been shown that benzo[a]pyrene will covalently bind to calf thymus DNA when incubated in the presence of rat liver microsomes (Gelboin, 1969). The binding is due to the formation of unknown metabolite(s) by the enzyme aryl hydroxylase. Thus, it would appear that rat liver microsomes are capable of producing metabolites of the carciogenic hydrocarbons which will covalently bind to DNA. For some reason the interaction between the activated metabolites and DNA does not occur in the rat liver system, and it may well be that the binding of carcinogen to soluble proteins in the cell sap which we have described, is important in preventing this interaction from being realized. The adsorption or non-covalent binding of methylcholanthrene to proteins of rat liver cell sap and in particular the pH 5 enzyme fraction is thus of interest and warrants further study.

We thank Miss G. Pye for excellent assistance with certain phases of the work. This work was supported by a grant from the Cancer Association of Rhodesia, Salisbury Branch.

### REFERENCES

Berwald, Y. and Sachs, L.—(1965) *J. natn. Cancer Inst.*, **35**, 641.
Bresnick, E., Liebelt, R. A., Stevenson, J. G. and Madix, J. C.—(1967) *Cancer Res.*, **27**, 462.
Brookes, P. and Lawley, P. D.—(1964) *Nature, Lond.*, **202**, 781.
Chauveau, J., Moule, Y. and Rouiller, C. H.—(1956) *Expl Cell Res.*, **11**, 317.
Diamond, L., Defendi, V. and Brookes, P.—(1967) *Cancer Res.*, **27**, 890.
Di Paolo, J. A. and Banerjee, M. R.—(1967) *Proc. natn. Acad. Sci. U.S.A.*, **58**, 123.
Gelboin, H.—(1969) *Cancer Res.*, **29**, 1272.
Gornall, A. G., Bardawill, C. S. and David, M. M.—(1949) *J. biol. Chem.*, **177**, 751.
Hawtrey, A. O., Schirren, V. and Dijkstra, J.—(1963) *Biochem. J.*, **88**, 106.
Heidelberger, C.—(1964) *J. cell. comp. Physiol.*, **64**, (Suppl. 1), 129.
Herrington, M. D. and Hawtrey, A. O.—(1969*a*) *S. Afr. J. med. Sci.*, **34**, 49.—(1969*b*) *Biochem. J.*, **115**, 671.
Huberman, E. and Sachs, L.—(1966) *Proc. natn. Acad. Sci. U.S.A.*, **56**, 1123.
Kirby, K. S.—(1956) *Biochem. J.*, **64**, 405.
Neville, D. M.—(1968) *Biochim. biophys. Acta.*, **154**, 540.
Prodi, G., Rocchi, P. and Grilli, S.—(1970) *Cancer Res.*, **30**, 1020.
Richardson, S. H., Hultin, H. O. and Green, D. E.—(1963) *Proc. natn. Acad. Sci. U.S.A.*, **50**, 821.
Scott-Burden, T. and Hawtrey, A. O.—(1969) *Biochem. J.*, **115**, 1063.
Sims, P.—(1966) *Biochem. J.*, **98**, 215.
Sporn, M. B. and Dingman, C. W.—(1966) *Nature, Lond.*, **210**, 531.
Wiest, W. G. and Heidelberger, C.—(1953) *Cancer Res.*, **13**, 250.